# Integration of conventional cell viability assays for reliable and reproducible read-outs: experimental evidence

**DOI:** 10.1186/s13104-018-3512-5

**Published:** 2018-06-22

**Authors:** Sukant Garg, He Huifu, Sunil C. Kaul, Renu Wadhwa

**Affiliations:** 1 DAILAB, DBT-AIST International Center for Translational and Environmental Research (DAICENTER), National Institute of Advanced Industrial Science & Technology (AIST), 1-1-1 Higashi, Tsukuba, 305-8565 Japan; 2 School of Integrative & Global Majors, University of Tsukuba, Tsukuba, 305-8577 Japan; 3 Graduate School of Life & Environmental Sciences, University of Tsukuba, Tsukuba, 305-0006 Japan

**Keywords:** QCV, Crystal violet, Clonogenicity, Quantitative–qualitative assay

## Abstract

**Objective:**

Short-term viability assays of cultured cells in 96-well plates are routinely used to determine the cytotoxicity or safety of drugs. These are often based on the formation of chromogen, generated selectively in viable cells. The innate problems of such short-term cell viability assays include (i) effect of drugs is determined by cell density (ii) some drugs have slow/gradual effect and hence may escape such assays, (iii) cell morphology that reveal significant hints to molecular signaling underlining the effect of drugs cannot be effectively captured, (iv) long-term effect on viability and clonogenic potential of cells cannot be determined and (v) herbal extracts often possess intrinsic color that interferes with spectrophotometer estimation. In light of the ease and importance of cell culture-based assessment of drug safety and cytotoxicity, we attempted to combine the conventional cell-based assays in a way that allows multiple readouts (quantitative and qualitative) from a single experiment, and avoids the drawbacks of color interference.

**Results:**

We have established and validated (using 16 types of cultured mammalian cells) a Quantitative and Qualitative Cell Viability assay in 12-well cell culture plates. It overcomes several shortcomings as discussed above and allows long-term observations on cell morphology and clonogenicity.

**Electronic supplementary material:**

The online version of this article (10.1186/s13104-018-3512-5) contains supplementary material, which is available to authorized users.

## Introduction

Cancer cells are characterized and distinguished from the normal cells by their potential to proliferate and grow in colonies [1], most usually treated with chemotherapy ranging from months to years [2]. However, most of the chemotherapeutic drugs are toxic to normal cells, and often result into resistance or recurrence [3]. Drug development involves complicated route that initiates with simple viability/cytotoxicity assays performed on cultured cancer and normal cells. Conventionally, these assays are performed in short-term (few hours) and rely on mitochondrial activity of viable cells, linked to reliable quantitative read-outs [4]. These have been proved very informative for extremely toxic drugs that cause sudden death of cells by apoptosis or autophagy. However, the drugs with slow, but useful, actions such as induction of growth arrest, senescence or differentiation require long term assessment [5–8].

In in vivo conditions, tumor cells grow from a single cell to a densely packed mass that determines the effect of drugs in several ways, therefore should be treated at sparse and dense clonogenic growth conditions. Furthermore, these cytotoxic compounds may cause morphologically accountable stress resulting into responses including sensitization or resistance [9], making cell morphology an important factor to consider cell response. Various metabolic-activity-based methods involving tetrazolium reduction and resazurin reduction have been described as reliable indicators of cell viability [10]. However, each lack universal application. Distinct factors limiting their use include concerns about cost, reagent-induced toxicity and operational interference [10, 11].

Large investments have been allocated towards identification and development of potential anticancer drug candidates [12]. National Institute of Health financial chart reported expenditure of about 5589 million US dollars in fiscal year 2016, out of which about 10% was spent on research [13–15]. These stakes are not justified if they do not sum up into successful clinical trials. American Cancer Society has already labeled cancer as an emerging epidemic [16]. This warrants investments in cancer drug discovery programs. Yet, the shrinking budget for medicinal research reflects lack of sophisticated instruments and personnel in small laboratories. We aimed and demonstrate here a technique designed to (i) allow evaluation of the effect of a drug on single cells developing into dense colonies, (ii) eliminate the interference of intrinsic color of the drug in viability measurements, and (iii) allow multiple readouts such as effect on morphology, clonogenicity and/or cytotoxicity in single experiment.

## Main text

### Materials and methods

#### Cell lines

All the cell lines purchased from JCRB, Japan [17] were carefully selected in order to include a variety from several tissues and diseases. Cells were cultured in a humidified 37 °C incubator with 5% CO_2_ following the supplier’s recommendation, as indicated in the Additional files 1 and 2.

#### Generation of standard curve

As presented in Additional files 2, 3 and 4, 0–80,000/well plated C6 cells were trypsinized, counted manually using cell counter (TC20™ Automated Cell Counter, Bio-Rad) in the 1st plate, and fixed with ice cold methanol: acetone (1:1) [18] in the 2nd plate, followed by staining, washing, air-drying, de-staining, and measurement of optical density in a 96-well plate at 570 nm using a spectrophotometer (Tecan Infinite 200^®^ Pro, Tecan Group Ltd., Mannedorf, Switzerland). Finally, a scatter graph was plotted using optical density against cell number to obtain *c* (y-intercept) and *m* (slope) values (Additional file 1).

#### MTT-based short- and long-term cell viability and microscopy

C6 or U2OS cells (1 × 10^3^/well) were plated in a 96-well plate and allowed to settle overnight, followed by treatment with DMEM supplemented with or without colored cytotoxic extract labelled CN-04 (*Cinnamomum verum* stem extract) or HA-05 (*Helicteres angustifolia* root extract). The control or extract-treated cells were incubated at 37 °C and 5% CO_2_. After 48 h, cell pictures were recorded at 40× magnification followed by washing with 200 µL PBS (twice) and replacement with fresh culture medium. 10 µL of MTT (M2128, Sigma-Aldrich) in phosphate buffered saline (PBS; 2 mg/mL) was added to each well and incubated at same conditions for 4 h. All the media was aspirated and replaced with 100% DMSO, and optical density was measured at 570 nm. Cell viability and standard deviation were calculated using Microsoft Office 2016^®^. Growth efficiency of live cells over long term (15–20 population doublings) in a 96-well plate was determined by the same method. 200–1000 C6 cells/well were plated in 96-well plates (4 sets) and allowed to settle overnight, followed by change in growth medium every alternate day. After every 48 h, cell viability was calculated in one set each. At the end, cell viability trend, standard deviation, slope equation and R^2^ values were collectively calculated.

#### Qualitative and Quantitative Cell Viability (QCV) assay

As presented in Additional files 3 and 4, C6 cells (100/well) were plated in a 12-well plate and incubated until the appearance of colonies (8–10 days) with regular change in the culture medium with/without the colored extract CN-04 (0.25–0.75%) or colorless compound CB-01 (Cucurbitacin B, 1 µM suspension in 100% DMSO, 0.5%) every alternate day. Cells and colonies were fixed using ice cold methanol: acetone (1:1) [18], followed by staining with crystal violet, washing, air-drying, phase contrast microscopy at 40–400× magnification, colony counting, de-staining, and measurement of optical density in a 96-well plate at 570 nm using the spectrophotometer. Colonies were averaged. Using the equation {*cell number *=(*OD *−* c*)/*m*}, average of long term cytotoxicity was obtained, where c and m are the y-intercept and slope values for C6 cells obtained in the generation of the standard curve section (Additional file 1).

#### Statistics

All the experiments were performed in triplicates. Statistical analysis was performed using GraphPad^®^ (2017) software, Inc. (California, USA), and depicted as * < 0.05, **0.01, and *** < 0.001. Unpaired t test was done using mean, standard deviation and the number of independent experiments.

### Results

**Fig. 1:**
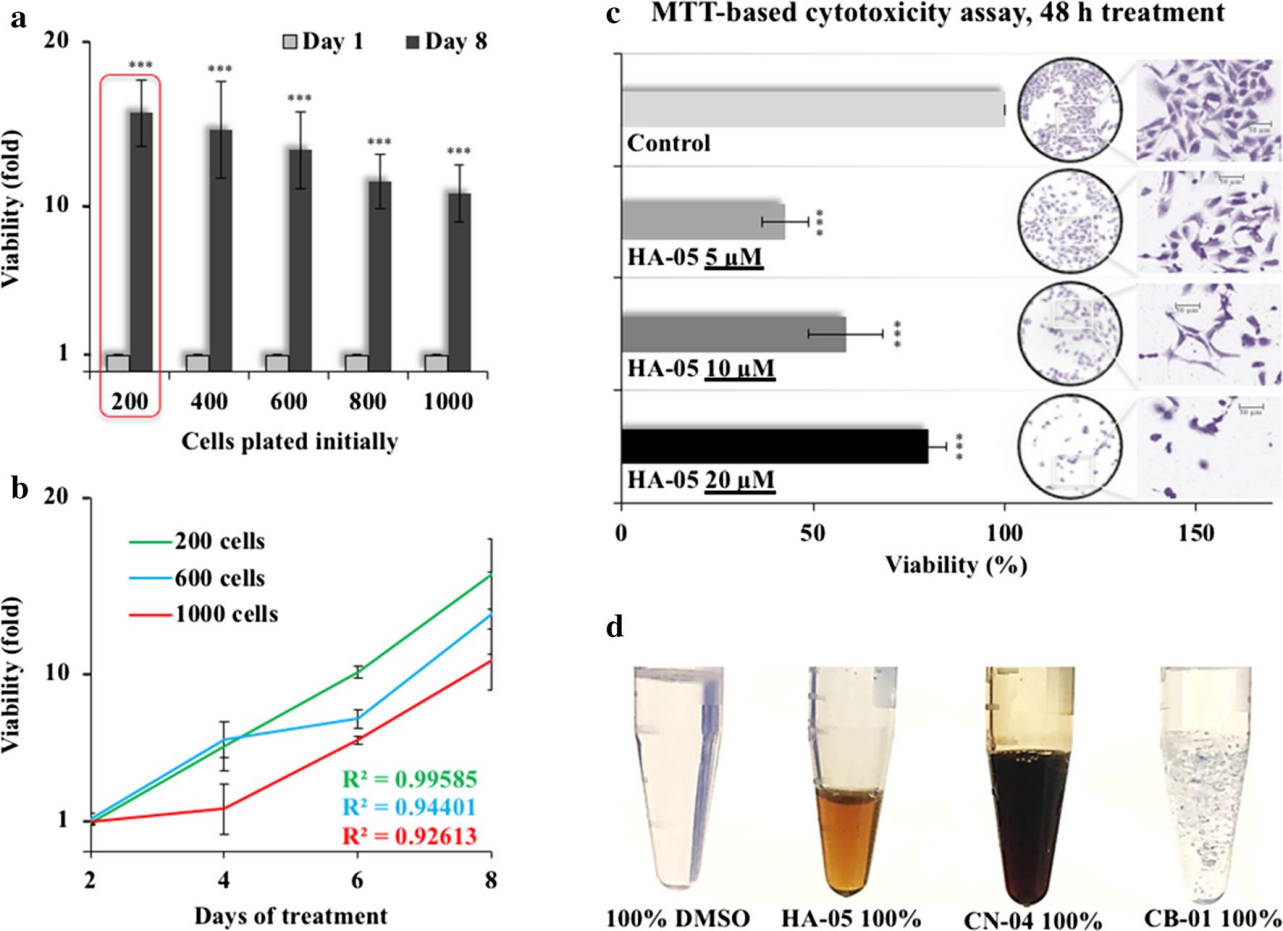
Interference of cell number and color of the test reagent in the cytotoxicity assays. **a** Cell viability after 8 days of culture in a 96-well plate well. **b** Cell growth pattern over 8 days in a 96-well plate, and **c** Cell viability after 48 h treatment with colored extract HA-05 (left) and cell pictures (right) against control recorded at ×40 magnification. **d** Images of the colored extracts and colorless compounds. Statistical analysis is depicted as * < 0.05, **0.01, and *** < 0.001

Growth characteristics of cells were determined for sixteen cell lines (Additional file 1). The optical densities were plotted to obtain standard curves and slope (y-intercept values). In our regular cell viability assays using MTT, we observed that the drug response is driven by cell density to a large extent. Hence, we examined the effect of cell density on growth or drug response in a time dependent manner. Due to fast growing properties (population doubling time ~ 10–12 h) that gives quick results, adequate for experimental validation, we selected rat glioma cells for the study. Cells plates at spare density formed macroscopically visible colonies in 7–8 days. Therefore, to enable the treatment of C6 cells individually as well as in colonies, a minimum of 8 days experiment was designed. This criterion also allowed us to monitor the effect of slow-acting compounds and add pragmatism to the actual in vitro/in vivo conditions to some extent. The experiments initiated with 200 C6 cells showed the most efficient growth in 8 days followed by cell density/adhesion dependent growth inhibition (Fig. 1a). Trend-line slope, y-intercept and R^2^ values showed that the 200 cells/well could qualify to be significant (criteria to qualify = R^2^ > 98%) (Fig. 1b). However, seeding of 200 cells per well in 96-well plate is considered low and likely to give high probability of experimental errors [10]. We next used plant extract HA-05 that possessed color. In independent 48 h MTT-based assays, we found that its color interfered with the optical density (Fig. 1c). Whereas low doses of the extract showed cytotoxicity by means of optical density measurement, the high doses showed increase in viability. Microscopic evaluation showed a clear dose dependent decrease in cell density as well as stressed morphology. Similar results were obtained with another colored compound CN-04 (Fig. 1d). Such errors in the readouts as a result of color caught our attention and urged remodeling of assays.

**Fig. 2:**
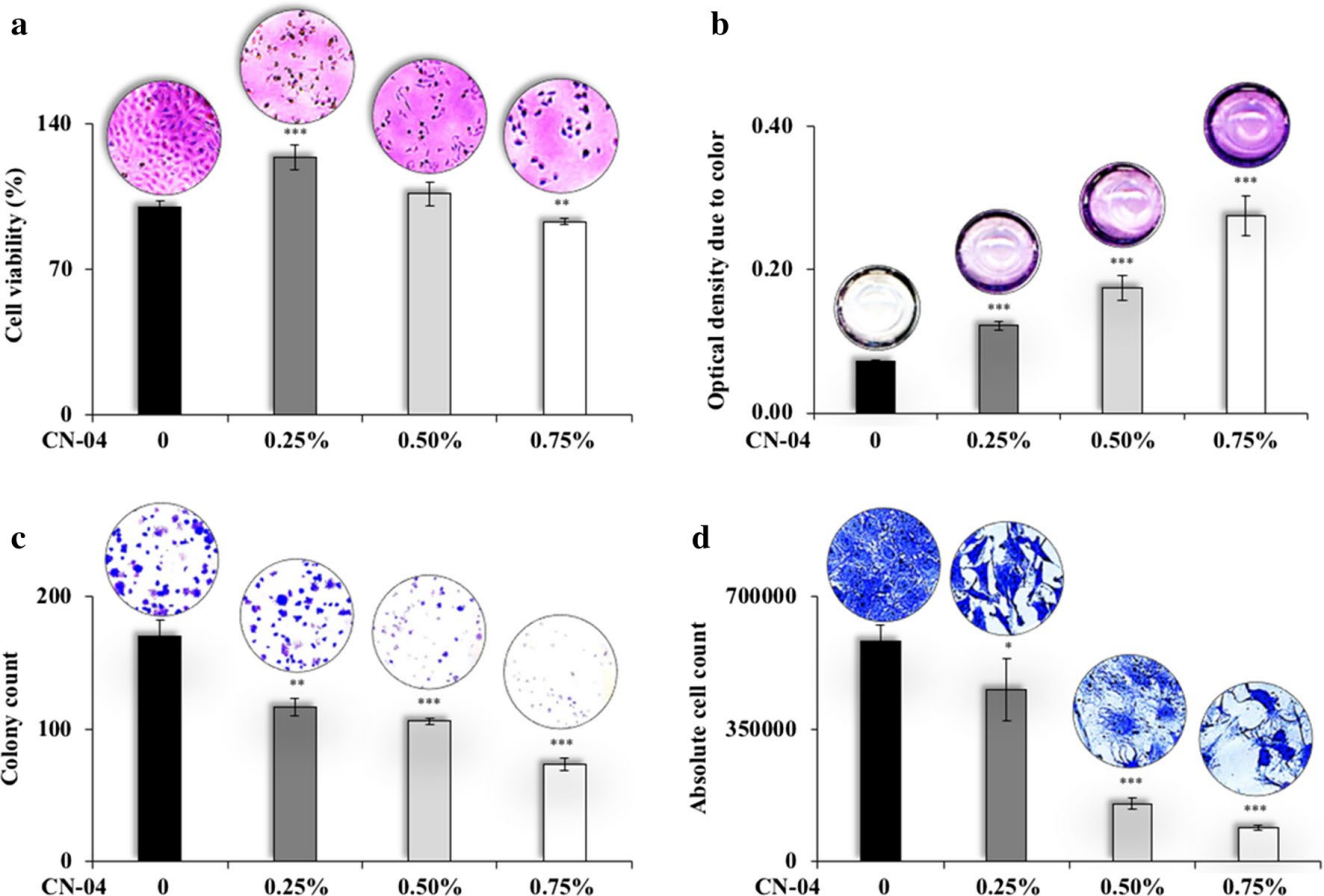
Determination of cytotoxicity of a colored reagent by conventional MTT assays, institution of QCV assay. **a** Viability of cells treated with the reagent for 48 h showed haphazard pattern, while the cell pictures recorded at ×40 magnification showed dose-dependent cytotoxicity. **b** Dose-dependent increase in optical density was observed as a result of color of the reagent. **c**, **d** Colony number and quantitative cell number determined by dissolving crystal violet stain, and the morphology of the cells recorded at ×40 magnification correlated with each other proportionately. Statistical analysis is depicted as * < 0.05, **0.01, and *** < 0.001

We recruited conventional MTT-based viability and our QCV assays on C6 cells treated with CN-04. MTT assay showed a discrepancy in the readings. Whereas cells treated with 0.25% CN-04 showed an increase in cell viability (Fig. 2a), microscopic observations showed cell death. In order to confirm the possibility of interference of the color, the extract (without cells) was incubated overnight in the same conditions and quantitated as in MTT assay. We found that the optical density from the wells corresponded directly with the dose of the extract (Fig. 2b). On the other hand, QCV determined cell viability (absolute cell count), morphological condition and the colony forming potential of the cells treated with CN-04 0.25–0.75% over 8-day period showed consistent results (Fig. 2c, d).

**Fig. 3:**
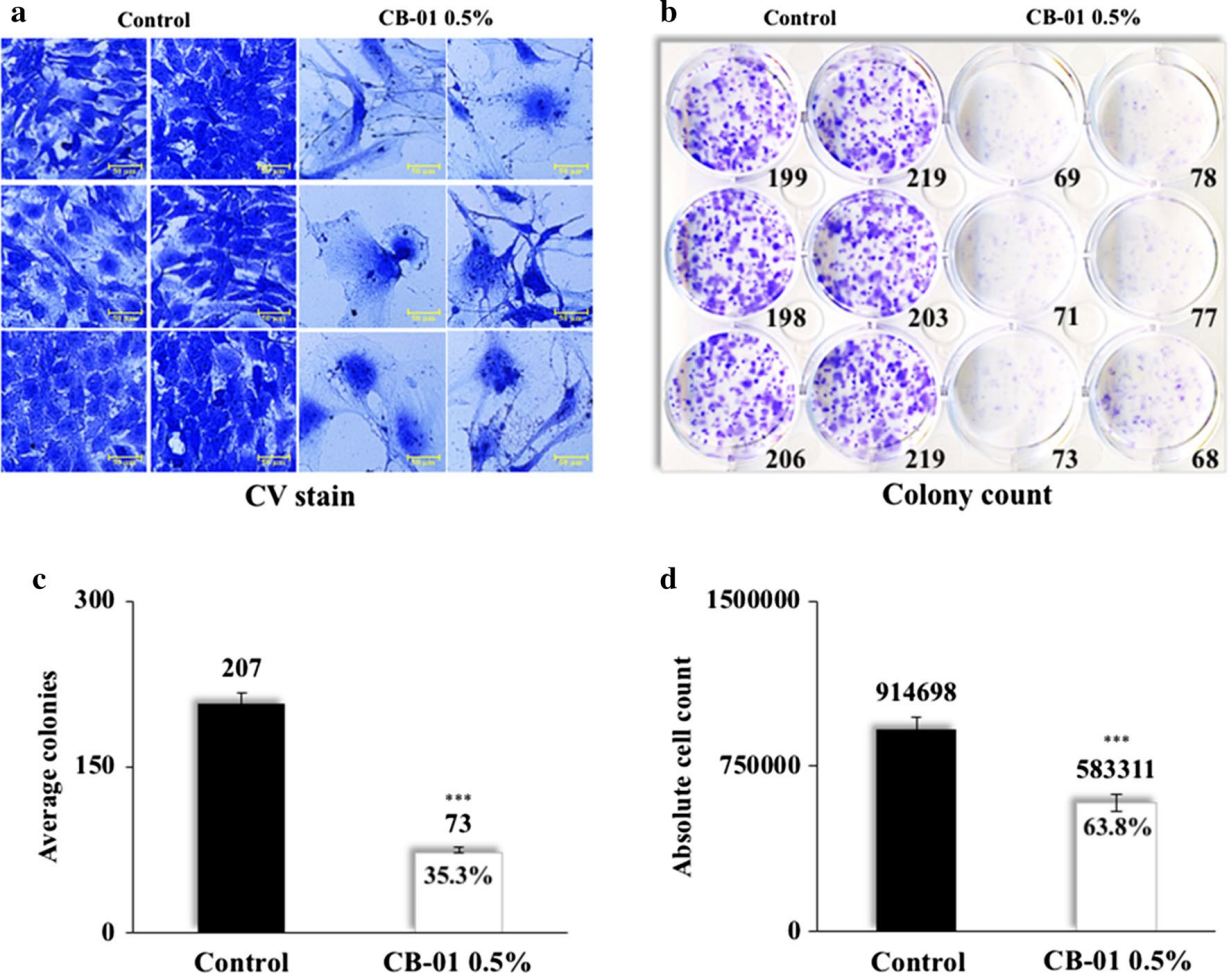
Validation of QCV assay: **a** Crystal violet stained cell pictures recorded at ×400 magnification at the end of 8 days treatment are shown. **b** Manually counted cell colonies in six variants of control and treated wells. **c** Quantified colony number, and **d** Quantified absolute cell count by dissolving crystal violet in de-staining solution and using slope equation for C6 cells. Statistical analysis is depicted as * < 0.05, **0.01, and *** < 0.001

In order to validate QCV assay for colorless agents, we performed colony formation assay using 0.5% of CB-01 (colorless cytotoxic compound). Besides the decrease in clonogenic potential, we confirmed considerable difference in the morphology of the treated cells against the control group (Fig. 3a–c). Optical density readout after de-staining revealed long-term quantifiable cell viability. Using the slope (m) and y-intercept (c) values given in Additional file 1, we calculated the absolute cell count in control and treated cells (Fig. 3d). Moreover, we found that the reduction in clonogenicity and viability in response to the treatment were not the same. While only ~ 35% colonies (Fig. 3c) remained after 8-days of treatment and culture, ~ 65% cells (Fig. 3d) in total survived, indicating plausible presence of individual, scattered and unperceivable cells within the well. Since these results suggest that the colony number and the absolute cell count are two independent entities, they must be noted separately.

In summary, we found that the QCV assay accounts for (i) cell count in direct proportional to the cell density and with no discrepancies due to the color of extract, (ii) additional readouts such as monitoring cell morphology and clonogenicity assessment making QCV assay more cost-effective, and (iii) convenient to measure the cell number and colony counts as independent records.

### Discussion

Conventional viability assays for cultured cells are the essential step in drug discovery protocols and may account for establishment of chemotherapeutic dose of the drugs in the clinics [19]. Most of these involve the application of intracellular metabolism altering reagents that are further complicated by drug and/or chromogen characteristics and interactions, such as crystallization, chemical interference, membrane permeability alteration, toxicity, and formazan fabrication with variable in vitro conditions with drug treatments [20–22]. Such hurdles have been implicated for many drugs [23], which may act slowly and demand time and dose dependent response over a relatively longer period [24]. To circumvent these difficulties, we assimilated the standard assays to yield a more precise and informative, quantifiable and reliable readout from a single experiment.

Based on the cell growth characteristics (Additional files 1 and 2), we chose C6 cells as they grew fast and yielded rapid validation. Each C6 cell (population doubling time ~ 12 h) was expected to multiply to about 65 × 10^3^ cells and make a small colony within 8 days. Therefore, to evaluate the effect of drug on single cells and colonies, the 96-well plates were considered inappropriate and experiment was performed in 12-well plates. We plated 100 cells per well in 12-well plates and subjected to treatment regime of 8 days with change in medium every alternate day. These criteria would allow the evaluation of slow-acting compounds rationalizing their activity in in vivo conditions to some extent. We found that the QCV was more consistent than the conventional MTT assay. We assumed the R^2^-value of more than 98% to be considered significant (more than 85% of standard deviation explained) [25, 26].

In QCV assay, it is possible to examine the effect of drugs in short as well as longer span of time in terms of viability and clonogenic potential of cells, irrespective of the cell size. Such readouts are more relevant to the cancer therapy regimens, especially those of natural origin and with slow mechanism of action—weeks to months [27]. QCV assay allows observations on cell morphology to envisage drug response characteristics [28–30]. Evaluation based on fixed cells account for absolute cell count instead of only the metabolically viable; multidimensional aspects depict economic performance [12–15, 31].

## Limitations

The present method may not be suitable for high-throughput screening.

## Additional files

**Additional file 1.** QCV standardization and determination of slope/y-intercept and R^2^ value in 16 cell lines.

**Additional file 2.** Cell lines, history of disease and conditions of incubation throughout the experiments.

**Additional file 3.** Schematic presentation of the protocol. **A.** Determination of standard curve and slope equation. **B.** QCV assay to determine cell viability, colony forming potential and cell morphology after long-term culture of cells.

**Additional file 4.** Step-by-step protocol of the QCV assay - determination of standard curve and slope equation, and three experiments turned into a single protocol.

## Abbreviations

QCV: Quantitative and Qualitative Cell Viability
JCRB: Japanese Collection of Research Bioresources Cell Bank
MTT: 3-(4,5-dimethylthiazol-2-yl)-2,5-diphenyltetrazolium bromide
CB: Cucurbitacin B
CV: crystal violet
