# Supplementary figures and images for: Integration of conventional cell viability assays for reliable and reproducible read-outs: experimental evidence
Source: BMC Res Notes. 2018 Jun 22;11:403. doi: 10.1186/s13104-018-3512-5 (PMC6013999; doi:10.1186/s13104-018-3512-5)

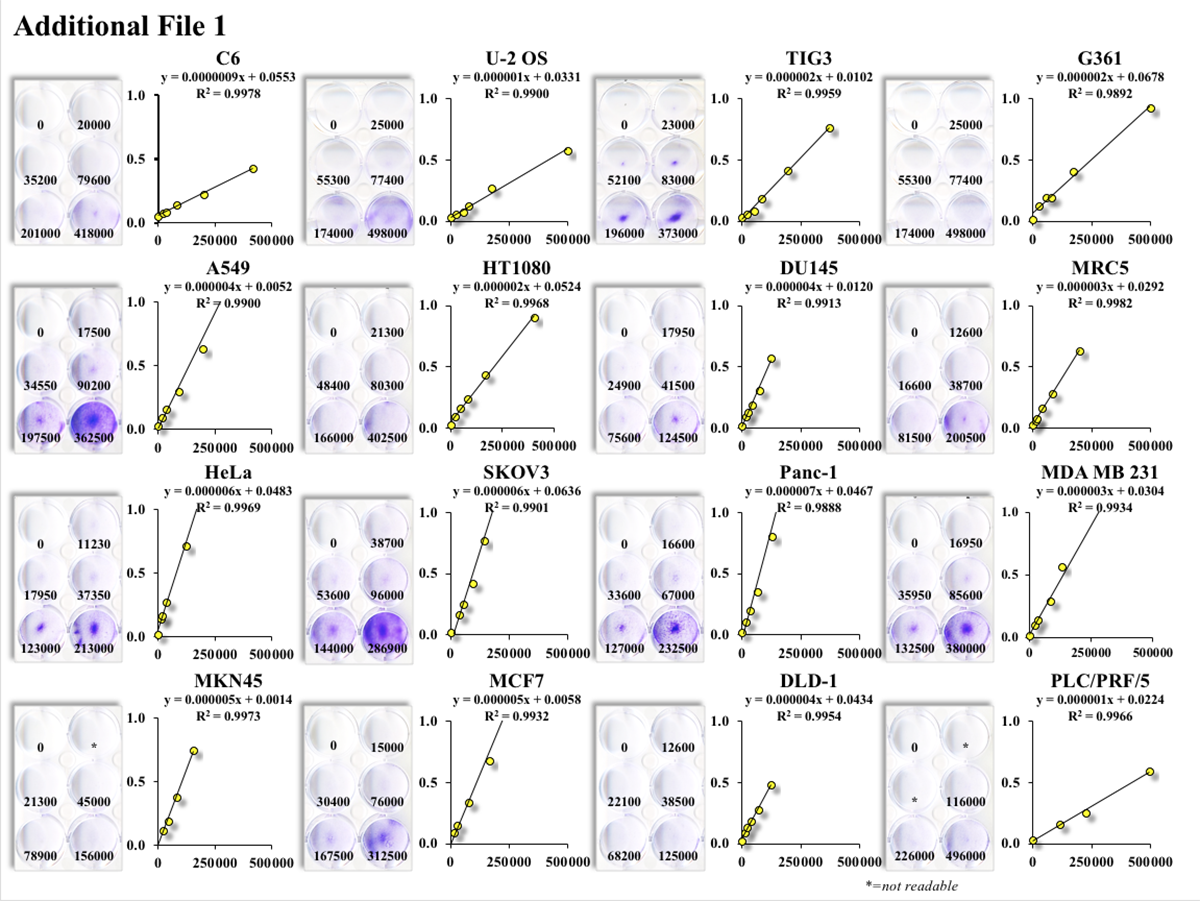

Supplement: Supplementary file 1 — Additional file 1. QCV standardization and determination of slope/y-intercept and R2 value in 16 cell lines. [file 13104_2018_3512_MOESM1_ESM.tif]

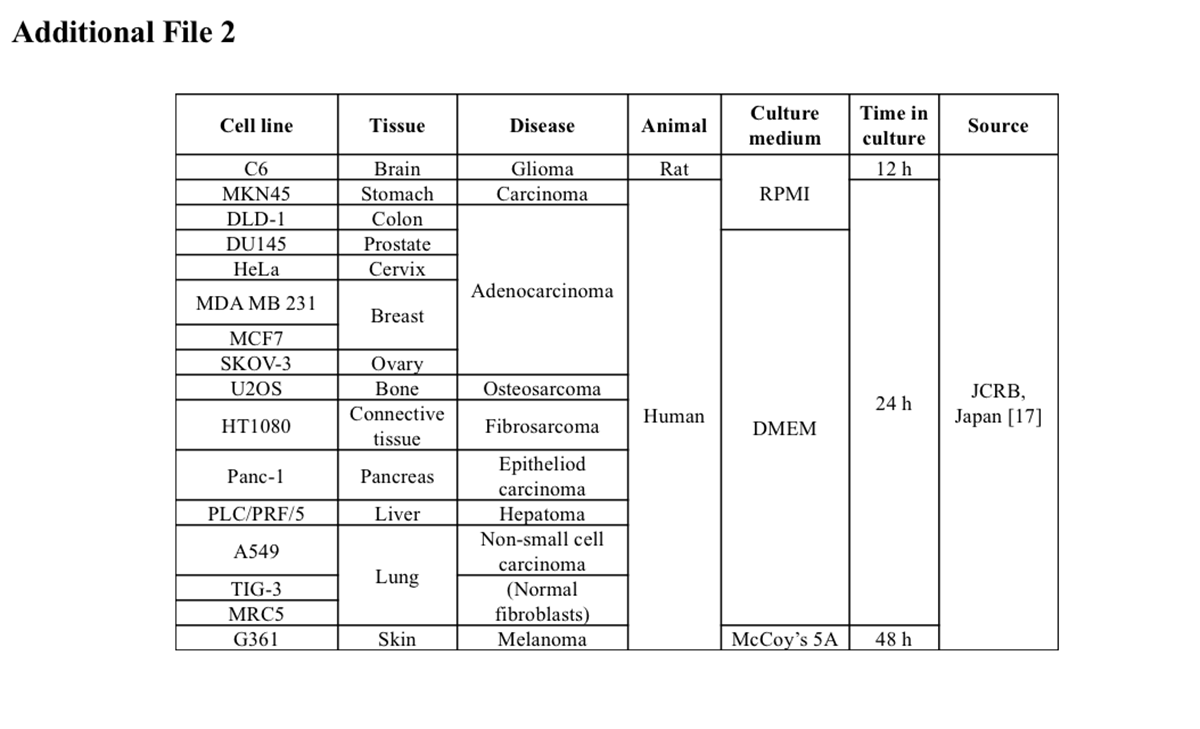

Supplement: Supplementary file 2 — Additional file 2. Cell lines, history of disease and conditions of incubation throughout the experiments. [file 13104_2018_3512_MOESM2_ESM.tif]

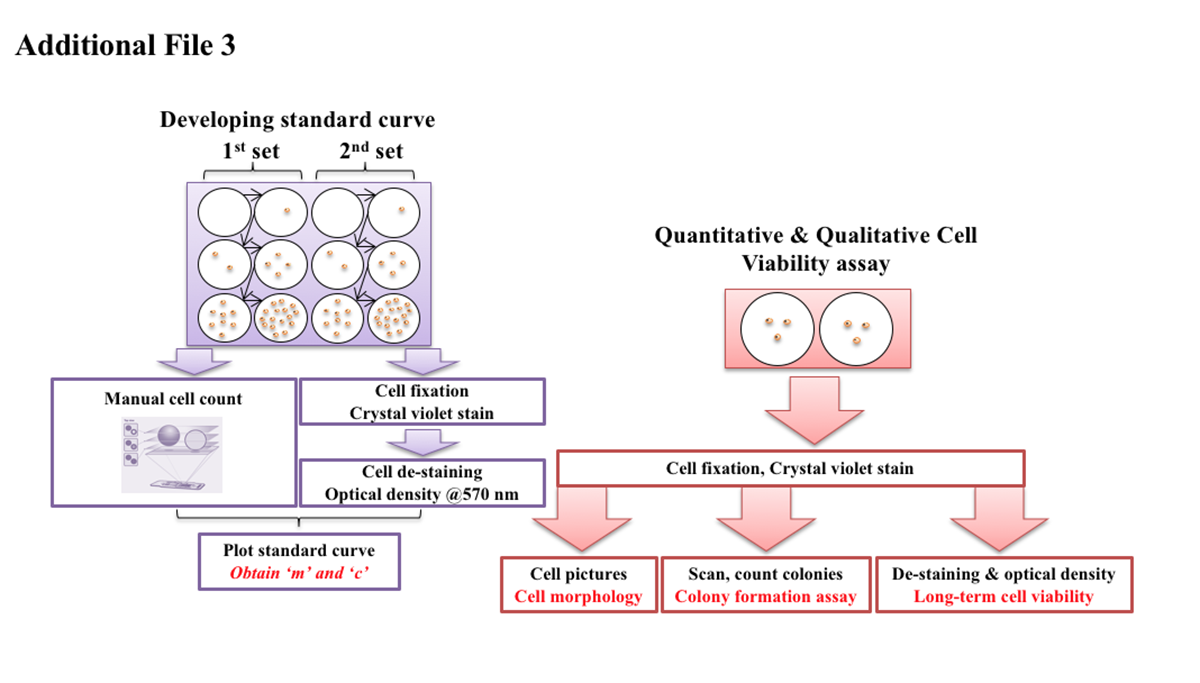

Supplement: Supplementary file 3 — Additional file 3. Schematic presentation of the protocol. A. Determination of standard curve and slope equation. B. QCV assay to determine cell viability, colony forming potential and cell morphology after long-term culture of cells. [file 13104_2018_3512_MOESM3_ESM.tif]

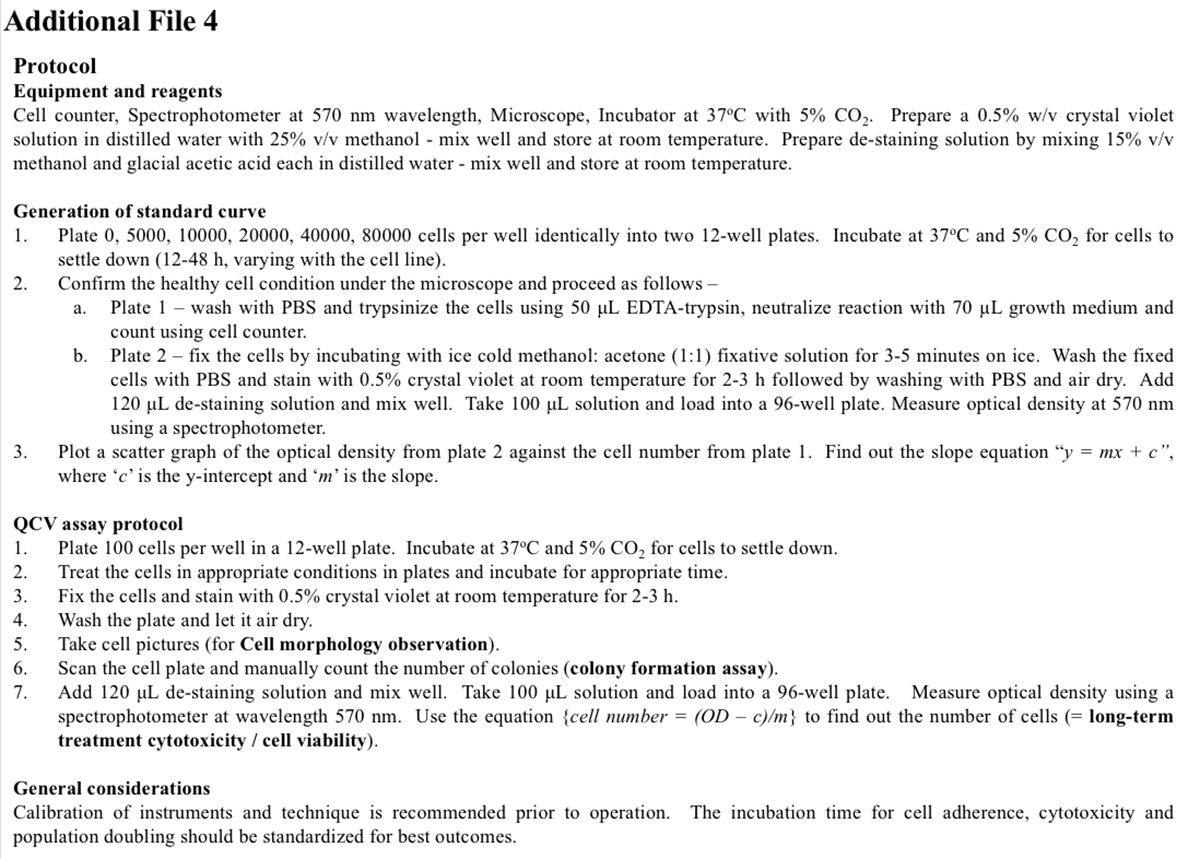

Supplement: Supplementary file 4 — Additional file 4. Step-by-step protocol of the QCV assay - determination of standard curve and slope equation, and three experiments turned into a single protocol. [file 13104_2018_3512_MOESM4_ESM.tif]
